# Dr. Edward Minturn

**Published:** 1858-11

**Authors:** 


IT is with profound regret that we record the death of our young friend, Dr. EDWARD MINTURN, which took place in this city on the 6th of September, from acute phthisis, in the twenty-eighth year of his age. Dr. M. was a highly educated gentleman, a conscientious and judicious practitioner, and honorable and exemplary in all his social relations. For some time he had been one of the physicians to the Philadelphia Dispensary, and had greatly endeared himself to his patients by his kindness, attention, and professional skill. He was also a zealous member of the Academy of Natural Sciences, and devoted most of his leisure time to scientific pursuits. His future was full of promise, and by his death the profession and science, equally with his family and friends, have sustained an irreparable loss.—*Medical News and Library*, October, 1858.

